# Is altitude friend or foe in unrepaired congenital heart disease? A case report

**DOI:** 10.1093/ehjcr/ytaf115

**Published:** 2025-03-24

**Authors:** Rebecca Fisher, Kali Hopkins, Krittika Pant, Arjun Kapoor, Ali N Zaidi

**Affiliations:** Department of Cardiology, Mount Sinai Fuster Heart Hospital, One Gustave L. Levy Place, New York, NY 10029, USA; Department of Cardiology, Mount Sinai Fuster Heart Hospital, One Gustave L. Levy Place, New York, NY 10029, USA; Department of Cardiology, Mount Sinai Fuster Heart Hospital, One Gustave L. Levy Place, New York, NY 10029, USA; Department of Cardiology, Mount Sinai Fuster Heart Hospital, One Gustave L. Levy Place, New York, NY 10029, USA; Department of Cardiology, Mount Sinai Fuster Heart Hospital, One Gustave L. Levy Place, New York, NY 10029, USA

**Keywords:** Patent ductus arteriosus, Pulmonary hypertension, Shunt, Congenital heart disease, Case report

## Abstract

**Background:**

Some unrestrictive congenital shunts put patients at high risk for developing irreversible pulmonary vascular disease if not closed in the first year of life. Living at high altitude also places patients at risk for developing pulmonary hypertension. There are anecdotal data that living at high altitude may be protective against the development of shunt-related pulmonary vascular disease.

**Case summary:**

A 20-year-old female from Ecuador was found to have a large patent ductus arteriosus (PDA) and pulmonary hypertension after a murmur was auscultated. She had been living at altitude until 6 months prior. She was found to have a large PDA, systemic pulmonary artery (PA) pressure, and an elevated pulmonary vascular resistance index (PVRi) (33 wu ∗ m^2^) with minimal shunting (Qp:Qs 1:1). Vasodilatory testing with inhaled nitric oxide (iNO) and 100% FiO2 showed continued systemic PA pressure but a dramatic decrease in PVRi (12.5 wu ∗ m^2^) with an increase in left-to-right shunt (Qp:Qs 2:1). Living at high altitude causes hypoxic vasoconstriction. This vasoconstriction, which is typically reversible on descent to sea level, may decrease the left-to-right shunt and the irreversible downstream vascular changes that would normally occur in the setting of an unrestrictive PDA. Given the significant acute drop of this patient’s vascular resistance with pulmonary vasodilator testing, she was started on oral pulmonary vasodilators. However, months later, the patient became increasingly symptomatic and repeat catheterization showed worsening cardiac function. Pulmonary vasodilators were stopped and the decision was made not to close the PDA.

**Discussion:**

Living at high altitude may be protective against the development of congenital shunt-related irreversible pulmonary vascular disease. However, in this case, it may not have been enough due to the complexity of anatomy.

**Learning points**

- Unrestrictive congenital shunts place patients at risk for developing irreversible pulmonary vascular disease often in later decades or with concomitant lung disease if not closed at a young age.
- Vasoconstriction from living at high altitudes may be protective against the development of irreversible pulmonary vascular disease. However, the individual’s nuanced congenital anatomy and time left unrepaired are important factors to consider when planning for intervention.

## Introduction

The ductus arteriosus is a normal foetal communication between the left pulmonary artery and aorta that should close in the first days of life. A patent ductus arteriosus (PDA), or ductus that remains open, occurs in ∼1 out of 2000 full-term live births.^1^ As the pulmonary vascular resistance drops in the first minutes to months of life, the left-to-right shunt (aorta to pulmonary artery) increases. The pressure and volume loads are related to the diameter of the PDA and the difference in vascular resistance between the systemic and pulmonary systems. A large PDA leads to high, unrestricted pressure and volume, which over time can lead to irreversible pulmonary vascular disease (i.e. Eisenmenger’s syndrome) often in later decades or with concomitant lung disease if not closed early. At high altitudes, a higher incidence of PDA is observed. One retrospective study observed that those at higher altitudes had larger PDA diameters and higher pulmonary artery pressure.^2^

Many people living at high altitudes develop pulmonary hypertension, though the biological mechanism appears to differ from the increased pressure and volume from shunt lesions and is usually reversible on descent to sea level. Through observational studies of populations at sea level and high altitude, differences in physiological and anatomic characteristics have been highlighted.^3^ In a normal newborn, there is right ventricular hypertrophy (RVH) and pulmonary arteriolar hypertrophy due to foetal circulation that regresses in the first months with post-natal adaptation. In those born and living at high altitudes, it is thought that low partial pressure of oxygen inhibits post-natal remodelling, leading to persistent muscularization of pulmonary arterioles. While individuals at sea level have a rapid decline in RVH and pulmonary artery (PA) pressure after birth, those at high altitudes have a more gradual decrease in RVH and PA pressure. In some patients, the elevated PA pressures persisting through life are thought to be secondary to the hypoxic pulmonary vasoconstrictor response. Descent to sea level usually allows for a reversal of the vasoconstriction with a normalization of PA pressure within months. In the case of a PDA or ventricular septal defect in a patient born and living at high altitude, the vasoconstrictor response will reduce the flow to the pulmonary vascular bed and may be protective against the development of shunt-related pulmonary vascular disease.

## Summary figure

**Table:** 

8/2023	Presented for bilateral slipped capital femoral epiphysis	II/VI machine-like murmur at the left upper sternal border auscultated	Echocardiogram completed
10/2023	Initial catheterization completed	Oral pulmonary vasodilators were started	
1/2024	Follow-up catheterization completed	Oral pulmonary vasodilators were stopped	Decision made not to close the PDA

## Case presentation

**Figure 1:**
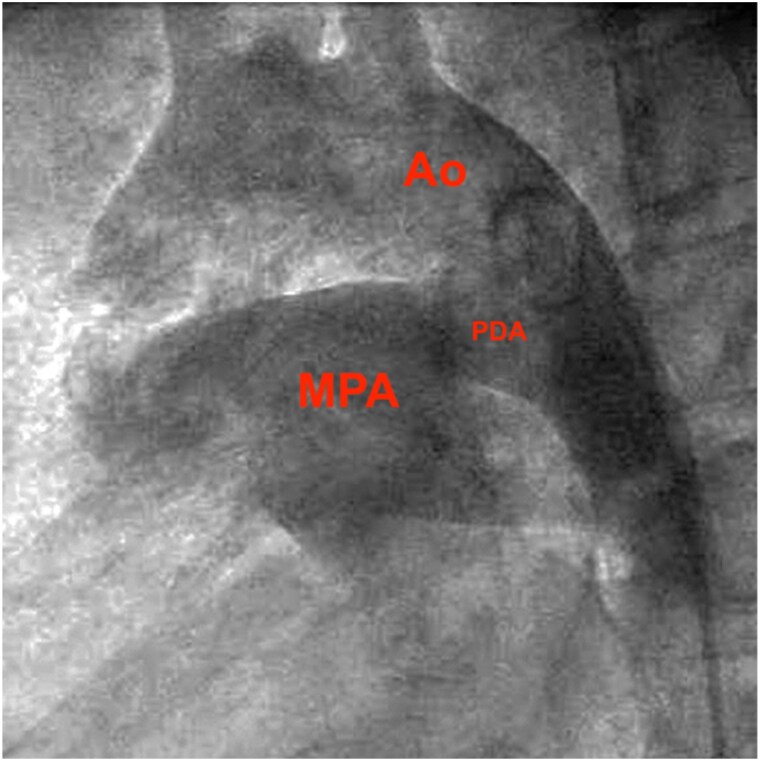
Lateral projection of an angiogram in the descending aorta showing the ductus arteriosus is roughly the same size as the descending aorta. Ao, aorta; MPA, main pulmonary artery; PDA, patent ductus arteriosus.

An asymptomatic 20-year-old female from Ecuador was found to have a large PDA after a II/VI machine-like murmur at the left upper sternal border was auscultated (*Summary figure*). Physical exam and labs were otherwise unremarkable. A transthoracic echocardiogram demonstrated a large PDA with bidirectional flow and systemic pulmonary hypertension. It also showed severe RVH and a moderately dilated left ventricle, though both with normal function. Cardiac MRI was completed with consistent findings. She was then referred for cardiac catheterization that confirmed a large PDA (18 mm diameter, *Figure 1*) and systemic-level pulmonary hypertension. Since birth, she had lived at 3100 m elevation before moving to sea level 6 months before presentation. Her past medical history was significant for hypothalamic-pituitary delayed-onset puberty and bilateral slipped capital femoral epiphysis.

On cardiac catheterization, there was systemic PA pressure and an elevated pulmonary vascular resistance index (PVRi) (33 wu • m^2^) with no net shunting (Qp:Qs 1:1). Vasodilatory testing with inhaled nitric oxide (iNO) and 100% FiO2 showed continued systemic PA pressure but a dramatic decrease in PVRi (12.5 wu • m^2^) with an increase in left-to-right shunting (Qp:Qs 2:1). Diagnostic balloon occlusion of the PDA on iNO and oxygen was incomplete due to the large PDA size. However, the PA pressure lowered to 75% systemic and the left-to-right shunt decreased (Qp:Qs 1.4:1) while PVRi remained unchanged (12.5 wu • m^2^) (*Table 1*).

**Table 1:** Cardiac catheterization measurements

	Initial cath	Initial cath	Initial cath	After 4 months on oral pulmonary vasodilator	After 4 months on oral pulmonary vasodilator
	FiO2 21%	FiO2 100% + iNO 80 ppm	FiO2 100% iNO 80 ppm PDA partially balloon occluded	FiO2 21%	FiO2 30%
RA	12 mmHg		13 mmHg	16 mmHg	19 mmHg
RPA	73% 119/64/91	92% 109/55/76	87% 83/35/58	80% 108/16/75	81% 105/34/75
RPCW	12 mmHg	20 mmHg	15 mmHg	28 mmHg	28 mmHg
LV	97% 119/10	100% 109/20	99% 112/17	94% 108/28	94% 105/28
dAO	97%	100%	99%	94%	94%
Qp:Qs	1:1	2.1:1	1.4:1	1.2:1	1.4:1
PVR	21 WU = 33 wu ∗ m^2^	7.8 WU = 12.5 wu ∗ m^2^	7.7 WU = 12.3 wu ∗ m^2^	7 WU = 11.4 wu ∗ m^2^	5.9 WU = 9.8 wu ∗ m^2^
SVR	21 WU = 33 wu ∗ m^2^	20.1 WU = 32 wu ∗ m^2^	17 WU = 27 wu ∗ m^2^	9.6 WU = 15.8 wu ∗ m^2^	8.5 WU = 14 wu ∗ m^2^

FiO2, fraction of inspired oxygen; iNO, inhaled nitric oxide; SVC, superior vena cava; RA, right atrium; RV, right ventricle; RPA, right pulmonary artery; RPCW, right pulmonary capillary wedge; LV, left ventricle; dAO, descending aorta; Qp, pulmonary flow; Qs, systemic flow; PVR, pulmonary vascular resistance.

Given the significant acute drop in this patient’s vascular resistance with pulmonary vasodilator testing, she was started on oral pulmonary vasodilators (tadalafil 40 mg daily and ambrisentan 5 mg daily) with planned repeat catheterization for re-evaluation in 4 months to determine the suitability of PDA closure.

**Figure 2:**
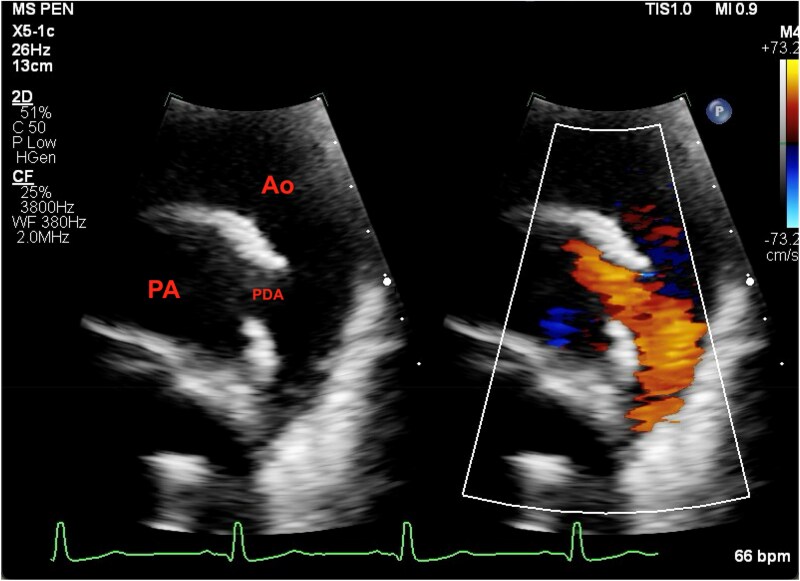
Echocardiogram after the patient received 4 months of pulmonary vasodilator therapies. Flow through the PDA demonstrates low-velocity left-to-right shunt.

Four months after the initiation of pulmonary vasodilators, a repeat catheterization showed that Qp:Qs was still 1.2:1 and that the left atrial pressure had increased significantly (to 28), consistent with diastolic dysfunction of the left ventricle in the setting of increased volume. The PA pressure remained systemic (as expected) given the unrestrictive nature of the ductus arteriosus. The pulmonary vascular resistance was lower than baseline, but still elevated (*Summary figure*, *Figure 2*). Pulmonary vasodilators were stopped and the decision was made not to close the PDA. She remains asymptomatic at this time.

## Discussion

This 20-year-old patient had a large unrestrictive PDA with evidence of high pulmonary vascular resistance. Given the significant acute drop in this patient’s vascular resistance with pulmonary vasodilator testing, it was hoped that a large component of the elevated vascular resistance was attributable to her living at altitude and that with her descent to sea level and the addition of pulmonary vasodilators, her pulmonary vascular resistance would fall to an acceptable level and allow for PDA closure.

Guidelines for PDA closure currently state that it can be considered in patients who have developed pulmonary arterial hypertension (PAH) with PVR ≥ 5 wu when there is still significant L-R shunt (Qp:Qs > 1.5), but it should be carefully considered.^4^ The next step of whether or not to close the PDA is a challenging decision. Per the guidelines, PDA closure is contraindicated (recommendation class III, harmful) for this patient as her pulmonary vascular resistance was still >2/3 systemic resistance.^4^ Even if the PDA were closed now, although the patient’s PA pressure would be subsystemic, it would not necessarily halt the progression of pulmonary vascular disease. If the PDA was closed and the pulmonary vascular resistance worsened, the PA pressure could become suprasystemic, leading to eventual failure of the right ventricle. At that point, she would likely become symptomatic with a shorter lifespan and require lifelong pulmonary hypertension medications. Leaving the PDA open results in systemic pulmonary arterial pressure but balanced systemic and pulmonary flow.

As her pulmonary vascular disease worsens, the pulmonary vascular resistance will likely supersede the systemic vascular resistance, leading to right-to-left shunting or Eisenmenger syndrome. Fortunately, the deoxygenated blood will go to the lower body preferentially through the PDA leading to differential cyanosis with cyanosis and clubbing in the feet which spares the upper body. She may benefit symptomatically from pulmonary vasodilators at that stage.

The idea of banding the PDA to reduce the pressure to the PA was rejected since the friability of PDA tissue risks catastrophic bleeding. Ligation of the PDA was also discussed with the creation of another small surgical shunt to limit the flow from the PDA and hopefully allow for pulmonary vascular remodelling on pulmonary vasodilators. The surgical shunt could act as a ‘pop-off’ if the pulmonary vascular resistance became suprasystemic. However, given the relatively low Qp:Qs at this point, the flow through a shunt would be small and likely lead to thrombosis of the shunt, rendering it ineffective. Closure of the PDA and creation of an atrial-level shunt was also considered; however, it would only be helpful as the RV failed. After careful consideration of the above and discussion with the patient, it was determined that her longevity and quality of life would be best served by not closing the PDA.^5,6^ Additionally, her pulmonary hypertension therapies were stopped due to medication side-effects and clinical worsening. Her clinical deterioration was likely secondary to increased left-to-right flow from pulmonary vasodilation. In a patient with an untreated shunt lesion, decreasing pulmonary vascular resistance will not change the pulmonary pressure but will increase the left-to-right shunt, which may exacerbate symptoms. It may also accelerate pulmonary vascular damage. Pulmonary vasodilators may be restarted when her PVR worsens and exceeds systemic resistance in which case she will become cyanotic in the lower extremities. At that point, pulmonary vasodilation is likely to offer symptomatic benefit.^7^ This case highlights the importance of evaluating each patient’s unique profile, and how in conjunction with current guidelines, closing the PDA would not have been in her best interest.

## Lead author biography


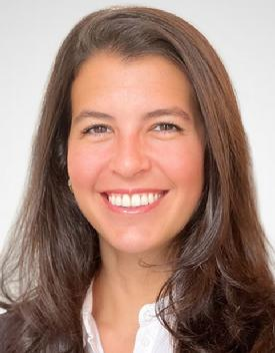


Rebecca Fisher is a third-year internal medicine resident at The Mount Sinai Hospital in New York City who will be pursuing a fellowship in cardiology next year.

## Data Availability

No new data were generated or analysed in support of this research.

## Notes

**Consent:** The patient provided consent in compliance with COPE guidelines.

**Funding**: No funding was received.
